# The Effectiveness of Neroli Essential Oil in Relieving Anxiety and Perceived Pain in Women during Labor: A Randomized Controlled Trial

**DOI:** 10.3390/healthcare10020366

**Published:** 2022-02-14

**Authors:** Cristiano Scandurra, Selene Mezzalira, Sara Cutillo, Rosanna Zapparella, Giancarlo Statti, Nelson Mauro Maldonato, Mariavittoria Locci, Vincenzo Bochicchio

**Affiliations:** 1 Department of Neuroscience, Reproductive Sciences and Dentistry, University of Naples Federico II, 80133 Naples, Italy; saracutillo99@gmail.com (S.C.); info@rosannazapparella.it (R.Z.); m.locci@unina.it (M.L.); 2 Department of Humanistic Studies, University of Calabria, 87036 Rende, Italy; selene.mezzalira@unical.it (S.M.); vincenzo.bochicchio@unical.it (V.B.); 3 Department of Pharmacy, Health and Nutritional Sciences, University of Calabria, 87036 Rende, Italy; g.statti@unical.it

**Keywords:** aromatherapy, neroli oil, anxiety, pain, labor, randomized controlled trial

## Abstract

Childbirth is a stressful and physically painful event in a woman’s life and aromatherapy is one of the most used non-pharmacological methods that is effective in reducing anxiety and perceived pain. This randomized controlled study aimed at determining the effect of neroli oil aromatherapy on anxiety and pain intensity perception in 88 women during labor, randomly assigned to either an intervention group (*n* = 44) or control group (*n* = 44). Anxiety and perceived pain were assessed through the visual analogue scale during the latent, early, and late active phases of labor. Data analyses included the *t*-test, Chi-square test, and repeated measures ANOVA. Perceived pain and anxiety in the group receiving aromatherapy were significantly lower than in the control group at all stages of labor (*p* < 0.05). Specifically, as the labor progressed, pain and anxiety increased in all participants, but the increase was milder in the experimental group than in the control group. The multiparas showed higher average anxiety scores, but not perceived pain, than the primiparas in all phases of labor (*p* < 0.05). Ultimately, neroli oil aromatherapy during labor can be used as an alternative tool to relieve anxiety and perceived pain in women during all stages of labor.

## 1. Introduction

During childbirth, fear and anxiety go hand in hand with anticipation and joy [1]. Indeed, childbirth is a stressful and physically painful event in a woman’s life, to the point that labor pain has been defined as one of the most severe types of human pain [2]. Perception of pain during labor is due to uterus’ contractions, uterine extension, and cervical dilation [3]. Inadequate pain management may affect diverse outcomes, such as psychological health, sexual functioning, or the infant–mother bond [4]. Previous studies stressed the association between pain and anxiety [5]. Anxiety activates the sympathetic nervous system releasing stress-related hormones (e.g., cortisol and adrenaline), which, as a consequence, may increase the severity of labor pain [6]. Therefore, it is imperative for midwives and obstetricians/gynecologists (Ob/Gyns) to find effective ways to relieve labor pain and anxiety.

Non-pharmacological methods such as relaxation techniques, massage, acupuncture, and aromatherapy are considered nowadays a promising area in midwifery thanks to their ease of use, low cost, and effectiveness [1,7,8,9,10]. Among them, aromatherapy represents one of the most used non-pharmacological methods for women in labor. It refers to the employment of the power of plant-sourced essential oils to treat and heal the individual’s body and psyche [11], and represents a strategy of care that utilizes essentials oils by massaging them into the skin, adding them to bath water, or inhaling their odor when added to a steam infusion [12].

Aromatherapy has been used to enhance women’s well-being during post-partum, as well as to facilitate mother–infant interactions [13]. It is often referred to as a useful means to alleviate anxiety and pain, thus fostering the individuals’ well-being [14]. Furthermore, aromatherapy has been shown to decrease anxiety and perceived pain during labor [15], as well as increase comfort and satisfaction [14,16]. Aromatherapy has been also successfully utilized jointly with massage to decrease stress and enhance immune function during pregnancy [12], as well as to reduce body tension and emotional stress [17].

Even though aromatherapy and essential oils such as orange scent, geranium, and lavender have been employed to reduce anxiety and perceived pain during labor [1,18,19,20], no studies have been carried out utilizing neroli oil to alleviate pain and anxiety during childbirth.

Neroli oil is extracted from the *Citrus aurantium* L. blossoms, commonly named bitter orange, which is a tree belonging to the *Rutaceae* family. It has antimicrobial and antioxidant properties [21], and has been shown to possess active constituents that play a significant role against inflammation, thus resulting useful for pain management [22]. Other therapeutic properties include sedative, calming, tonic, cytophylactic, aphrodisiac, anti-depressant, and antispasmodic action [23]. Most importantly, neroli oil can be utilized as an anxiolytic [24]. Therefore, neroli oil is frequently used for medicinal purposed, in particular for treating gastrointestinal disorders, tachycardia, and rheumatism, for minimizing central nervous system disorders [25], and as a sedative [26]. *Citrus Aurantium L.* flowers produce the orange blossom water, also utilized for therapeutic purposes [27]. Originally employed as a cardiac stimulant, for carminative purposes, and to help babies fall asleep, this water has been suggested to be useful in detoxification programs or when quitting addiction habits such as smoking [23]. Besides the aromatic water, the distillation of sour orange flowers produces neroli, a rare aromatic oil that contains a fragrance and represents the core of one of the world’s most used perfumes, “eau de cologne,” which is also used in pharmacy as a flavoring agent [21], as well as in some medicines approved by the American Food and Drug Administration. 

Based on these premises, this study aimed at determining the effect of neroli oil aromatherapy on anxiety and pain intensity perception in a group of women in labor.

## 2. Materials and Methods

### 2.1. Essential Oil Chemical Characterization

Neroli essential oil was purchased by Gya Labs. The essential oil chemical characterization was performed on 100% pure oil. In the final product, instead, the essential oil was used in a 5% formulation.

The investigated essential oil was characterized through a Hewlett-Packard 6890 gas chromatograph equipped with a 100% dimethylpolysiloxane SE-30 capillary column (30 m length, 0.25 mm in diameter, 0.25 µm film thickness), coupled with a Hewlett Packard 5973 mass spectrometer. A programmed temperature ranging from 60 to 280 °C, with a rate of 16 °C/min was used; the analysis was performed by using helium (0.00167 cm/s linear velocity) as carrier gas.

Essential oil constituents were identified by matching the obtained spectra with those listed in the Wiley 138 mass spectral library.

### 2.2. Study Design and Procedures

This was a prospective, interventional, non-pharmacological, and randomized controlled study, with a repeated-measure design.

Participants were randomly distributed in the experimental or in the control group according to a randomization with a 1:1 ratio obtained through a web-based computer system (randomization.com). The pregnant women in the control group received only routine prenatal care, which included emotional support from a midwife, the ability to take free positions during labor, massage and/or the application of hot packs in the lumbosacral area. The pregnant women in the intervention group received routine prenatal care plus the aromatherapy with vapor diffusion.

The essential neroli oil was diffused continuously through an aroma diffuser and using standard concentration at four drops of aroma oil per 300 mL of diffused water. The aromatherapy lasted the whole time of labor. Anxiety and pain intensity perception were assessed during 3 stages of labor: the latent phase (cervical dilatation of 3–4 cm), early active phase (cervical dilatation of 5–7 cm), and late active phase (cervical dilatation of 8–10 cm).

To better promote the spread of the active ingredients of neroli oil, a water (50%) and alcohol (32%) based formulation was made in which a percentage of 5% of the essential neroli oil was added. The formulation was completed by a phenolic antioxidant agent, BHT or butylhydroxytoluene, used at 0.1% and finished with the addition of PEG-40 hydrogenated castor oil, which has emulsifying functions, and propylene glycol, a carrier that makes the fragrance more lasting.

All participants provided written informed consent. The study was approved by the Ethical Committee of Psychological Research of the University of Naples Federico II (protocol number 2/2021; date of approval: 9 February 2021), designed with respect of the principles of the Declaration of Helsinki, and conducted following the EU General Data Protection Regulation. The clinical trial was retrospectively registered on the Deutsches Register Klinischer Studien (n° DRKS00027563).

### 2.3. Participants and Sample Size

Participants were recruited from May to October 2021 at the prenatal clinic of the University Public Hospital Federico II of Naples, which is also an obstetric emergency department.

All pregnant women, aged between 18 and 40 years, with a low-risk full-term pregnancy (between the 37th and 42nd week of amenorrhea) undergoing labor and with the fetus in cephalic presentation were included in the study. Pregnant women with maternal and/or fetal pathologies, subjected to drug induction to labor, or who had resorted to epidural analgesia, were excluded from the study.

A statistical power analysis was performed for sample size estimation through G*Power program (Heinrich Heine University, Düsseldorf, Germany). Based on parameters used by Tanvisut et al. [28], the effect size was set at 0.05, the *α* at 0.05 (two-tailed), and the power at 90%. Results indicated that a sample size of at least 42 participants for each group was needed.

**Figure 1:**
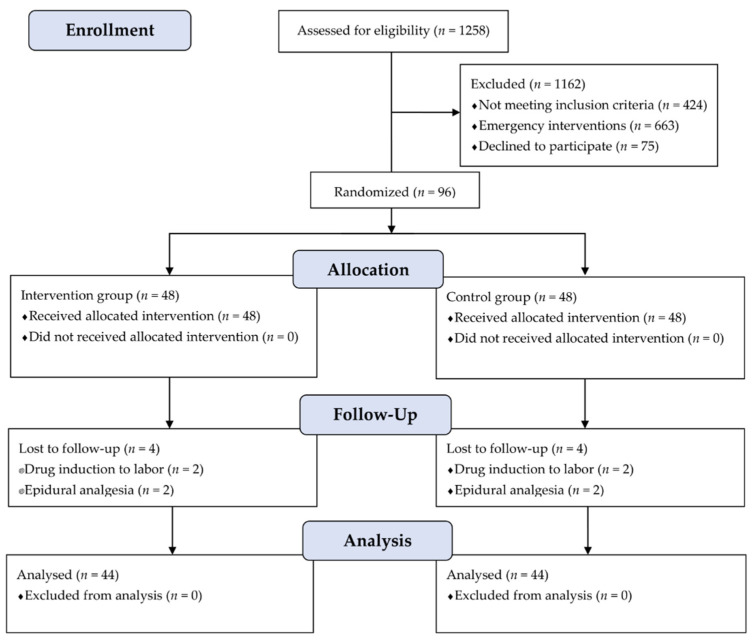
CONSORT diagram of study participants in control and intervention groups.

During the study period, 1258 women were admitted to the delivery room at the hospital. Among them, a total of 96 women met the inclusion criteria. They were randomly assigned to either the intervention group (*n* = 48) or control group (*n* = 48). Four women for each group were then excluded because they needed to take drugs to induce labor or to undergo epidural analgesia. Thus, analyses were conducted on 44 women in the experimental group and 44 in the control group. The CONSORT flow diagram is shown in Figure 1.

### 2.4. Measures

#### 2.4.1. Clinical and Demographic Information

Clinical and demographic variables assessed in this study included age, parity (primiparas vs. multiparas), duration of labor, and the Apgar index.

#### 2.4.2. Anxiety

Anxiety was assessed through two measures: the Visual Analogue Scale for Anxiety (VAS-A [29]; Italian adaption by Facco et al. [30]) and the State-Trait Anxiety Inventory Form Y (STAI-Y [31]; Italian adaption by Pedrabissi and Santinello [32]).

VAS-A is a measure assessing perceived levels of anxiety which is particularly effective in those situations where answering many questions may be burdensome for participants, as well as it is for women during labor. The VAS-A is a line 10 centimeters in length with zero representing “not at all anxious” and 10 “very anxious”. Participants are asked to mark their subjective anxious status on a visual scale by putting a cross. Different studies demonstrated the validity of the VAS-A (for a review, see Rossi and Pourtois [33]). VAS-A was assessed during latent, early active, and late active phases of labor.

STAI-Y is a measure consisting of 20 items that assess transitory feelings of tension, worry, and nervousness at a given moment. The answer options range from 1 (“not at all”) to 4 (“very much so”), with higher scores indicating greater state anxiety. In our sample the values of Cronbach’s alpha at the moment of the recruitment and immediately after the birth were 0.81 and 0.80, respectively. This measure was administered at the moment of the recruitment (i.e., before the childbirth), and immediately after the childbirth.

#### 2.4.3. Pain Intensity

Pain was assessed through the Visual Analogue Scale (VAS [34]; Italian adaptation by De Benedittis et al. [35]), a widely used scale assessing the perceived intensity of pain. The VAS is a continuous unidimensional scale comprised of a horizontal line 10 centimeters in length with zero representing “no pain” and 10 “worst pain”. Participants are asked to mark their perceived pain intensity on a visual scale by putting a cross. Different studies demonstrated the validity of the VAS (for a review, see Bijur et al. [36]). As the VAS-A, even the pain VAS was assessed during latent, early active, and late active phases of labor.

### 2.5. Statistical Analyses

Statistical analyses were conducted using SPSS version 27 (IBM, Armonk, NY, USA) and setting the level of significance at 0.05. 

First, Student’s *t*-test for continuous variables or chi-square (*χ*^2^) for frequencies were used to evaluate any socio-demographic or clinical differences between experimental and control group and assess whether the two groups were comparable.

Then, Student’s *t*-test was performed to evaluate the differences between the experimental and control groups in the mean scores on perceived pain and anxiety during different stages of labor. The effect size was calculated with Cohen’s *d* [37] (small effect size = 0.01, medium effect size = 0.06, and large effect size = 0.14). 

Finally, a repeated measures ANOVA was performed to evaluate the effect of “Study Group” (experimental vs. control), “Time” (i.e., the three phases of labor), and “Childbirth Group” (primiparas vs. multiparas) on pain and anxiety. The effect size, in this case, was calculated with Cohen’s *η*^2^ [37] (small effect size = 0.01, medium effect size = 0.06, and large effect = size 0.14). 

## 3. Results

### 3.1. Gas Chromatography–Mass Spectrometry (GC-MS) Analysis

Major constituents of neroli essential oil were investigated through gas chromatography–mass spectrometry (GC-MS) analysis (Table 1). The monoterpene Linalool represents the most abundant compound in the essential oil composition (10.70 ± 0.55 %), followed by anthranilic acid, limonene, α-terpineol, and geranil acetate, with value percentage of 6.43 ± 0.60, 3.91 ± 0.12, 3.31 ± 0.15, and 3.21 ± 0.23, respectively. Other compounds, such as 4-carene and α-ocimene, were found in trace amounts.

### 3.2. Participants’ Characteristics

Fifty-one participants were primiparas (26 in the experimental group and 25 in the control group) and 37 multiparas (18 in the experimental group and 19 in the control group). No statistical differences in sample terms were detected (*χ*^2^ = 0.04, *p* = 0.84).

The average age of the participants was 31 years (*SD* = 5.64) in the experimental group and 32.11 years (*SD* = 5.60) in the control group, and the difference was not significant (*t* = −0.43, *p* = 0.67).

The mean duration of labor was 2.47 hours (*SD* = 1.51) in the experimental group and 2.32 hours (*SD* = 1.59) in the control group, and the difference was not significant (*t* = 0.21, *p* = 0.84).

The averages of the Apgar index at 1 min (experimental group: *M* = 8.00, *SD* = 0.82; Control group: *M* = 8.11, *SD* = 1.27; *t* = −0.23, *p* = 0.82) and at 5 min (experimental group: *M* = 8.90, *SD* = 0.32; Control group: *M* = 9.00, *SD* = 0.50; *t* = −0.53, *p* = 0.60) did not differ significantly between groups.

The absence of statistically significant differences on the variables reported made the groups comparable.

### 3.3. Effect of Neroli Oil Aromatherapy on Pain Intensity

The results showed that participants undergoing aromatherapy had lower perceived pain intensity than participants in the control group at all stages of labor (Table 2).

Repeated measures ANOVA revealed that the main effect of the “Study Group” (experimental vs. control) was significant (*F* = 7.55, *p* = 0.01, *η*^2^ = 0.32), indicating that there was an overall difference in the mean pain scores of the experimental group compared to those of the control group, with a large effect size. Similarly, the “Time” effect (i.e., the three phases of labor) was also significant and with a large effect size (*F* = 6.98, *p* = 0.003, *η*^2^ = 0.30). On the contrary, no significant effect was found for the “Childbirth Group”, indicating that the results obtained did not depend on being primiparas or multiparas.

**Figure 2:**
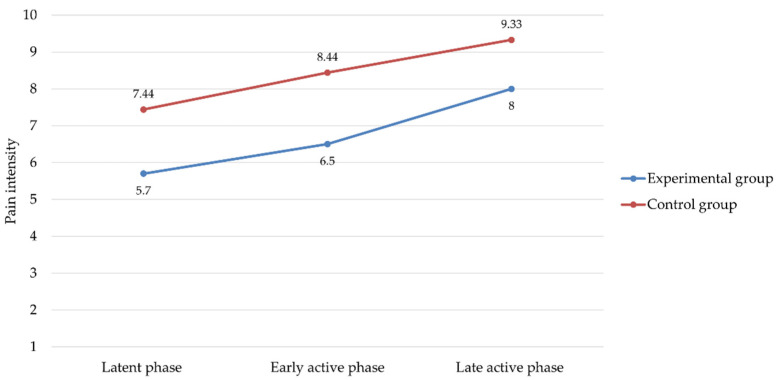
Changes in pain intensity scores along the stages of labor in experimental and control group.

Overall, as shown in Figure 2, as labor progressed, women in both groups perceived pain as getting stronger, but the increase was milder in the experimental group than in the control group.

### 3.4. Effect of Neroli Oil Aromatherapy on Anxiety

The results relating to anxiety assessed through the VAS-A were similar to those obtained for pain. Indeed, they showed that participants undergoing aromatherapy perceived lower levels of anxiety than participants in the control group at all stages of labor (Table 3).

**Figure 3:**
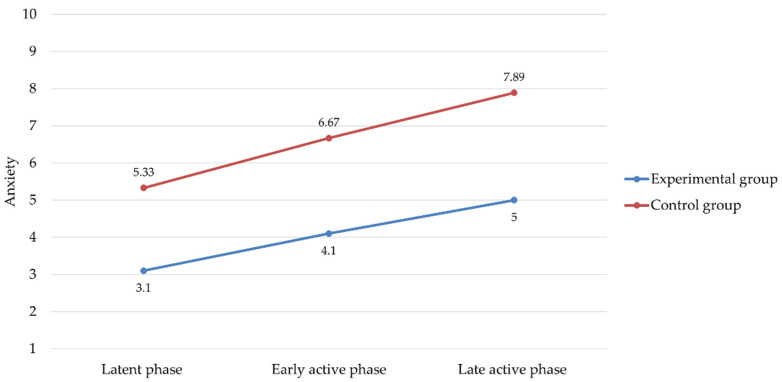
Changes in anxiety scores measured with the Visual Analogue Scale for Anxiety along the stages of labor in experimental and control group.

Repeated measures ANOVA revealed that the main effect of the “Study Group” (experimental vs. control) was significant (*F* = 11.41, *p* = 0.004, *η*^2^ = 0.42), indicating that there was an overall difference in mean anxiety scores reported by the experimental group compared to those of the control group, with a large effect size. Similarly, the “Time” effect (i.e., the three phases of labor) was also significant and with a large effect size (*F* = 7.66, *p* = 0.014, *η*^2^ = 0.32). However, as opposed to the results concerning pain intensity, in this case even the effect of the “Childbirth Group” (primiparas vs. multiparas) on anxiety was significant and with a large effect size (*F* = 16.19, *p* = 0.001, *η*^2^ = 0.50). Specifically, the multiparas showed higher average anxiety scores than the primiparas in all phases of labor, as follows: latent phase (primiparas: *M* = 2.91, *SD* = 2.12; multiparas: *M* = 5.87, *SD* = 1.12; *t* = −3.59, *p* = 0.002), early active phase (primiparas: *M* = 4.09, *SD* = 2.47; multiparas: *M* = 7.00, *SD* = 1.07; *t* = −3.11, *p* = 0.006), and late active phase (primiparas: *M* = 5.00, *SD* = 2.65; multiparas: *M* = 8.25, *SD* = 1.67; *t* = −3.05, *p* = 0.007). Again, as shown in Figure 3, with the progress of labor, anxiety increased in all participants, but the increase was milder in the experimental group than in the control group.

Finally, with regard to state anxiety assessed through the STAI-Y, it clearly emerged that the average anxiety scores measured before labor did not differ between the experimental and control groups, while differed significantly after childbirth, indicating that neroli oil aromatherapy had a positive effect on anxiety (Table 4).

## 4. Discussion

The current randomized controlled study was aimed at evaluating the effectiveness of neroli essential oil aromatherapy in relieving anxiety and perceived pain in women in labor. Results showed that neroli oil aromatherapy significantly and positively impacts women’s experience of perceived pain and anxiety during labor, representing a further confirmation of the effectiveness of non-pharmacological methods in making the childbirth a less stressful experience.

Specifically, our findings showed that neroli oil reduced women’s perception of pain and anxiety, which appeared less intense than in the women that did not receive aromatherapy treatment. Specifically, as the labor progressed, pain and anxiety increased in all participants, but the increase was milder in the experimental group when compared to the control group. Furthermore, since the average anxiety and perceived pain scores measured before labor did not differ between the experimental and control groups, while differed significantly after childbirth, we can conclude that neroli oil aromatherapy had a positive effect on anxiety and perceived pain. These findings confirm the results obtained in previous studies using other essential oils [16,28,38,39]. For instance, the use of essential oils in aromatherapy, thanks to their validated analgesic, anti-inflammatory, calming, and relaxing effects, has been proven to alleviate physical and emotional disorders in cancer patients [40]. This makes it reasonable to infer that aromatherapy represents a helpful alternative method for anxiety and pain control [41,42,43].

A second finding showed that the mean anxiety scores were higher in the multiparas than in the primiparas in all stages of labor. This might be explained by taking into account the role that previous experience might play in pregnant women’s experience [44]. Provided that childbirth is a physically painful experience, we might hypothesize that having already gone through labor can be a predisposing factor for greater expectations of perceived pain, therefore explaining the differences in perceived anxiety in the two groups (i.e., primiparas vs. multiparas). However, this is a speculative and hypothetical explanation, as other studies have shown that primiparas experience more anxiety than multiparas during labor [44,45,46,47]. Thus, future studies should collect data about the quality of previous childbirth experiences in multiparas and assess whether negative experiences can affect anxiety during labor.

Findings should be read in light of some limitations. First, this study assessed only one mode of aromatherapy and one essential oil, and did not compare different techniques of aromatherapy administration or other essential oils. Future research should replicate this study assessing whether other techniques of aromatherapy and other essential oils are more or less effective than that used in this study. Second, the stressful condition under which participants had to answer the questionnaires may have confounded their responses to the pain and anxiety assessment. However, we tried to overcome this intrinsic limitation by administering not stressful and easy to use questionnaires (i.e., VAS and VAS-A). Third, we did not collect information about previous childbirth experiences or any other experience (e.g., previous negative medical experiences) that could have affected anxiety. 

Despite these limitations, the findings obtained in the current study point to the fact that available non-pharmacological remedies such as aromatherapy are effective in relieving pain and anxiety in women during the most difficult phase of pregnancy, that is, labor and childbirth [48]. They also represent a viable alternative to a strict medicalization of labor [49], providing midwifes and Ob/Gyns with natural methods that can be easily used [10]. The relevance of our results also consists in the fact that when pain and anxiety are less severe, labor progresses more easily and with less difficulties. Therefore, neroli oil can and should be used as an alternative tool to relieve anxiety and perceived pain in women during all stages of labor.

## 5. Conclusions

Neroli oil aromatherapy during labor significantly impacts women’s experience of perceived pain and anxiety, which seem to be reduced in their severity during all stages of labor. Since neroli oil is a non-pharmacological remedy, which is efficacious in relieving perceived pain and anxiety in women during labor and childbirth, it represents an extremely useful alternative to pharmacological drugs. In fact, neroli oil aids in the progress of labor by decreasing perceived pain and anxiety, thus rendering labor and childbirth easier and less problematic.

## Figures and Tables

**Table 1:** Gas chromatography–mass spectrometry (GC-MS) analysis.

N.	Compound ^a^	Rt ^b^	RAP ^c^
1	α-Pinene	6.33	2.72 ± 0.09
2	Camphene	6.63	0.12 ± 0.01
3	β-Pinene	7.20	0.85 ± 0.07
4	β-Myrcene	7.47	1.50 ± 0.10
5	3-Carene	7.81	1.85 ± 0.14
6	Isocineole	7.88	0.26 ± 0.01
7	Limonene	8.16	3.91 ± 0.12
8	4-Carene	8.24	Tr ^d^
9	α-Ocimene	8.40	Tr
10	Linalool	9.25	10.70 ± 0.55
11	1-p-menthol	9.83	0.47 ± 0.02
12	Acetophenone	10.27	0.13 ± 0.01
13	α-terpineol	10.43	3.31 ± 0.15
14	Citronellol	10.86	1.73 ± 0.09
15	Anthranilic acid	10.95	6.43 ± 0.60
16	Geraniol	11.09	1.52 ± 0.20
17	1,4-dimethyl-4-vinylciclohexene	11.29	1.30 ± 0.08
18	Fenchyl acetate	11.35	0.49 ± 0.02
19	Indole	11.66	0.21 ± 0.01
20	Geranyl acetate	12.15	3.21 ± 0.23
21	Tridecanol	12.80	2.28 ± 0.18
22	Nerolin	13.44	1.47 ± 0.16
23	Nerolidol	13.63	0.29 ± 0.01
24	Farnesol	14.62	1.20 ± 0.05

Notes: ^a^ Major compounds listed in order of elution from SE30 MS column; ^b^ Retention time (as min); ^c^ Relative area percentage (peak area relative to total peak area in total ion current (TIC) %); ^d^ Tr: Traces percentages < 0.1%. Data are expressed as mean ± standard deviation (*n* = 3) of 3 independent experiments.

**Table 2:** Comparisons between experimental and control groups on pain intensity during the stages of labor.

	Experimental Group (*n* = 44) *M* (*SD*)	Control Group (*n* = 44) *M* (*SD*)	*t*	95% CI	*d*
Latent phase	5.70 (1.42)	7.44 (1.33)	−2.75 *	−3.08, −0.41	1.26
Early active phase	6.50 (1.27)	8.44 (1.42)	−3.15 **	−3.25, −0.64	1.44
Late active phase	8.00 (1.56)	9.33 (1.12)	−2.11 *	−2.66, −0.01	0.98

Notes: *M* = mean; *SD* = standard deviation; *t* = Student’s *t*-test; CI = confidence intervals; *d* = Cohen’s d. * *p* < 0.05; ** *p* < 0.01.

**Table 3:** Comparisons between experimental and control groups on anxiety measured with VAS-A during the stages of labor.

	Experimental Group (*n* = 44) *M* (*SD*)	Control Group (*n* = 44) *M* (*SD*)	*t*	95% CI	*d*
Latent phase	3.10 (2.13)	5.33 (1.94)	−2.38 *	−4.21, −0.25	1.09
Early active phase	4.10 (2.68)	6.67 (1.22)	−2.63 *	−4.63, −0.50	1.23
Late active phase	5.00 (3.02)	7.89 (1.45)	−2.61 *	−5.23, −0.55	1.22

Notes: VAS-A = Visual Analogue Scale for Anxiety; *M* = mean; *SD* = standard deviation; *t* = Student’s *t*-test; CI = confidence intervals; *d* = Cohen’s d. * *p* < 0.05.

**Table 4:** Comparisons between experimental and control groups on anxiety measured with STAI-Y before and after the childbirth.

	Experimental Group (*n* = 44) *M* (*SD*)	Control Group (*n* = 44) *M* (*SD*)	*t*	95% CI	*d*
Before the childbirth	2.07 (0.15)	2.08 (0.18)	−0.04	−0.16, 0.16	1.09
After the childbirth	2.01 (0.06)	2.32 (0.19)	−4.69 ***	−4.44, −0.17	1.23

Notes: STAI-Y = State–Trait Anxiety Inventory Form Y; *M* = mean; *SD* = standard deviation; *t* = Student’s *t*-test; CI = confidence intervals; *d* = Cohen’s d. *** *p* < 0.001.

## Data Availability

The data and materials that support the findings of this study are available from the corresponding authors upon reasonable request.
